# Nanosized food additives impact beneficial and pathogenic bacteria in the human gut: a simulated gastrointestinal study

**DOI:** 10.1038/s41538-018-0030-8

**Published:** 2018-12-04

**Authors:** Svenja Siemer, Angelina Hahlbrock, Cecilia Vallet, David Julian McClements, Jan Balszuweit, Jens Voskuhl, Dominic Docter, Silja Wessler, Shirley K. Knauer, Dana Westmeier, Roland H. Stauber

**Affiliations:** 1 Department of Nanobiomedicine/ENT, University Medical Center of Mainz, Langenbeckstrasse 1, 55131 Mainz, Germany; 2 Department of Molecular Biology II, Centre for Medical Biotechnology (ZMB), University Duisburg-Essen, Universitätsstraße 5, 45117 Essen, Germany; 3 Department of Food Science, University of Massachusetts Amherst, Amherst, MA 01003 USA; 4 Institute for Organic Chemistry, University of Duisburg-Essen, Universitätsstraße 5, 45117 Essen, Germany; 5 Department of Microbiology, Paris-Lodron University of Salzburg, A-5020 Salzburg, Austria

**Keywords:** Pathogens, Infection

## Abstract

Nanotechnology provides the food industry with new ways to modulate various aspects of food. Hence, engineered nanoparticles (NPs) are increasingly added to food and beverage products as functional ingredients. However, the impact of engineered as well as naturally occurring NPs on both commensal and pathogenic microorganisms within the gastrointestinal tract (GI) is not fully understood. Here, well-defined synthetic NPs and bacterial models were used to probe nanoparticle–bacteria interactions, from analytical to in situ to in vitro. NP–bacteria complexation occurred most efficiently for small NPs, independent of their core material or surface charge, but could be reduced by NPs’ steric surface modifications. Adsorption to bacteria could also be demonstrated for naturally occurring carbon NPs isolated from beer. Complex formation affected the (patho)biological behavior of both the NPs and bacteria, including their cellular uptake into epithelial cells and phagocytes, pathogenic signaling pathways, and NP-induced cell toxicity. NP–bacteria complex formation was concentration-dependently reduced when the NPs became coated with biomolecule coronas with sequential simulation of first oral uptake and then the GI. However, efficient NP adsorption was restored when the pH was sufficiently low, such as in simulating the conditions of the stomach. Collectively, NP binding to enteric bacteria may impact their (patho)biology, particularly in the stomach. Nanosized-food additives as well as naturally occurring NPs may be exploited to (rationally) shape the microbiome. The information contained in this article should facilitate a “safe by design” strategy for the development and application of engineered NPs as functional foods ingredients.

## Nanotechnology: how nanoparticles impact the gut microbiome

Engineered or naturally occurring nanoparticles could potentially affect the bacteria in the gut. A study led by Dana Westmeier and Roland Stauber from University Medical Center of Mainz, Germany probed the nanoparticle–bacteria interactions in situ. They found that NP–bacteria complex occurred most efficiently for small NPs, independent of their core material or surface charge. The complex formation affected the (patho)biological behavior of both the NPs and bacteria, particularly under conditions that simulate the stomach. The result shows that both engineered and naturally occurring nanoparticles could be exploited to shape the gut microbiome. The study can offer guidelines for future development and application of nanoparticles in food industry.

## Introduction

The applications of nanoparticles (NPs) in agriculture, biotechnology, foods, personal care products, and medicine are rising exponentially, which means that humans, animals and the environment are increasingly being exposed to NPs.^1–3^ In the food industry, engineered NPs are being used as lightening agents, colors, nutrient delivery systems, or antimicrobial agents, and may therefore be ingested by humans as part of nanoenabled foods and beverages.^4^ The gastrointestinal fate of NPs most likely differs considerably from that of larger particles because of their higher surface area, greater Brownian motion, and ability to penetrate biological barriers, such as the mucus layer or epithelium of eukaryotic cells, more easily.^4,5^ It is, therefore, important to ensure that any nanoenabled food ingredients are safe for application in foods.

The human body coexists with multitudes of microorganisms that may be either beneficial or detrimental to human health. In particular, the complex microbial ecosystems present within the human gastrointestinal tract (GI) tract are known to profoundly shape intestinal host physiology and are major mediators of the impact of diet on the host’s metabolic and disease status.^3,6,7^ Moreover, dysbiosis and reduced diversity of the commensal gut microbiota appear to be associated with inflammatory and metabolic diseases.^8,9^ As this complex microbial ecosystem coevolves in a mutualistic relationship with the human host, changes in human lifestyle and diet are an important evolutionary selection pressure on the gut microbiome.^10^ Exposure to ingested NPs, even for a short time, may modify the composition and diversity of the commensal microbiome, and therefore impact human health and well-being.^3,11^ However, researchers have only begun to explore the complex interaction of NPs with microbes and its potential beneficial or detrimental biological consequences.^11,12^

When discussing NPs in food science, we distinguish between naturally occurring “soft” and “hard” NPs, such as the casein micelles found in milk or the oil bodies present in nuts and beans, from engineered NPs, which are intentionally added for a specific functional purpose, unintentionally generated during food processing, or accidently taken up from the environment.^3,4^ Intentionally added engineered NPs provide the food industry with new approaches to improve the quality, shelf life, safety, and healthiness of foods.^4^ NPs can be incorporated into a food or beverage as a delivery system for colors, flavors, preservatives, nutrients, and nutraceuticals and/or to modify the optical, rheological and stability properties of the products.^3,4,11^ Even if only trace amounts of such substances are present in the end product, the safety of all NPs, either purposely added^4,13^ or generated during the production process^3,4,11^ should be considered. As food is by far the most important substance to interact with the human body in terms of quantity and frequency, the potential adverse or even beneficial impact of ingested food-grade NPs should be investigated and mechanistically understood to reduce potential risks or even exploit our knowledge to improve human health.^3,14^ Moreover, the interaction of NPs with enteric (a)pathogenic bacteria should be studied to determine if NPs might exhibit additional antimicrobial activity and/or are capable of modulating the microbiome via different mechanisms.^3,15–17^

Besides interacting with the bacteria of the healthy gut microbiota, ingested NPs may also interact with any pathogenic bacteria taken in through the nose or mouth.^3^ Thus, NPs found in food do not only interact with food associated microbes, such as probiotics, but also with potentially infectious microbes.^3,18,19^ One representative is the spiral bacterium *Helicobacter pylori*, whose unique ecological niche is the human stomach. *H. pylori* gastritis is etiologically associated with chronic gastritis, peptic ulcers, primary gastric B-cell lymphoma, and gastric carcinoma. Chronic inflammation caused by infection with *H. pylori* is one of the strongest risk factors for gastric adenocarcinoma, a leading cause of cancer-associated death worldwide.^3,20–22^ *H. pylori*-associated diseases are determined by bacterial pathogenic factors, including cytotoxin-associated gene A (CagA) and its associated type IV secretion system (T4SS).^23,24^ In general, T4SS are diverse nanomachines that vary in function and complexity across bacterial species.^25,26^ CagA is injected via the T4SS into host cells where it can be rapidly phosphorylated by kinases leading to altered cell signaling, proliferation, cytokine production, and changes in cell polarity and motility.^22,26^ In addition to antibiotics and anti-inflammatory drugs, probiotic microorganisms, specific diets, and certain food additives are being investigated for their potential to protect against *H. pylori* infections.^27,28^ Clearly, there is an urgent need to better understand the complex influence of the physiological and physicochemical microenvironment of the gut on the behavior of commensal and pathogenic bacteria in the GI tract. However, only very few reports have previously studied the (patho)biological consequences of NP-microbe interactions.^3,29^ In particular, the impact of nonbactericidal, food-derived NPs on the fate of enteric bacteria and the cellular microenvironment of the GI tract have not been studied. As this type of knowledge is critical for the safe and efficacious application of nanotechnology in foods, we have carried out a series of carefully designed experiments to elucidate the underlying principles. This was achieved by using a range of NPs with well-defined characteristics to simulate those currently or potentially utilized in the food industry as functional ingredients.

## Results

### In situ self-assembly of NP–bacteria hybrid structures

NPs’ physicochemical characteristics (Supplementary Fig. S1a) clearly define their behavior and (patho)biological activity.^18,30,31^ Hence, it is important to study representative and well-characterized model NPs of varying composition, size, shape, and surface functionalization (Table 1). Information obtained from studying these model NPs, combined with that obtained from studying actual industrially utilized nanosized food additives, will allow one to correlate specific NP characteristics to (patho)biological effects. In our study, NPs were thoroughly characterized by a series of independent analytical methods, including electron microscopy, dynamic light scattering (DLS), and ζ potential measurements (Table 1). The NPs examined in this study include industrial products manufactured in large quantities, international reference materials, NPs isolated from food, and well-defined NPs produced especially for research purposes. This selection provides a good model system for NPs currently used in the food sector as well as those that might be used in future applications.^3,4,32^

**Table 1:** Nanoparticle characterization

Nanoparticles	TEM diameter in dry state±s.d. (nm)	Hydrodynamic diameter in water±s.d. (nm)	Zeta potential±s.d. (nm)	Bacteria binding
***Silica–NPs***				
Si_30_	31.6 ± 5.8	33 ± 7	−15 ± 2	+SEM/EDX/AFM
Si_30C_	27.2 ± 3.8	28 ± 7	−18 ± 2	+SEM
Si_30N_	30.2 ± 6.8	31 ± 10	−10 ± 0.5	+SEM
Si_140G_	140.8 ± 8.0	142.4 ± 6	−20 ± 3	+FM*
Si_140N_	144.8 ± 8.0	148 ± 10	−18 ± 2	+SEM
Si_140C_	129.0 ± 4.0	133 ± 10	−28 ± 3	+SEM
Si_140_	141 ± 6.0	141 ± 6	−21 ± 3	+SEM/EDX/AFM
Si_R_	30.6 ± 6.8	33.6 ± 8	−14 ± 2	+FM*/SEM
Si_G_	30.8 ± 6.4	34.0 ± 7.6	−15 ± 2	+FM*
Si_B_	30.7 ± 6.2	33.0 ± 5.6	−14 ± 1	+FM
SiP 22 S	135 ± 11.2	143 ± 14.3	−27 ± 2	+SEM
SiP 350	45 ± 5.6	52 ± 7.3	−23 ± 2	+SEM
SiP D 17	100 ± 9.8	112 ± 12.3	−18 ± 1	+SEM
Levasil CS40-213P	22 ± 3.1	23 ± 2.4	−20 ± 3	+SEM
Levasil CS50-34P	55 ± 7.8	122 ± 8.4	−30 ± 4	+SEM
*Polymer–NPs*				
POSi_RC_	9.1 ± 1.8^1^	14.9 ± 0.09	−32 ± 2	+FM*
POSi_RN_	10.2 ± 1.9^1^	15.7 ± 0.09	+ 24 ± 5	+FM*
POSi_RPEG_	10.4 ± 1.7^1^	22.1 ± 0.09	−14 ± 1	−FM*
POSi_RPEtO_	11.8 ± 2.0^1^	26.0 ± 0.19	−5 ± 1	−FM*
*Metal-based NPs*				
Ag	10.3 ± 2.2^1^	12.4 ± 0.5	−43 ± 3	+SEM/EDX
CuO	55.2 ± 3.6^1^	488.3 ± 12	−4.5 ± 0.5	+SEM
FeO_G_	194.0 ± 12.0^1^	200 ± 8	−20 ± 1	+FM/SEM
Endorem contrast agent	n.d.	106 ± 15	−46.8 ± 4	+SEM/EDX
Fe_2_O_3_@SiO_2_-1	35.4 ± 0.3 (Fe_2_O_3_: 4.3 ± 0.4)	209 ± 36.2	−27.0 ± 0.6	+SEM
*Carbon nanomaterials*				
CN_NM400_	11.0 ± 3.0 × 846 ± 446^1^	n.d	n.d	+SEM
CN_NM401_	67.0 ± 26.2 × 4,048 ± 2,371^1^	n.d	n.d	+SEM
CN_NM402_	11.0 ± 3.0 × 1,372 ± 836^1^	n.d	n.d	+SEM
Carbon black	175 ± 8.8 (Pore size: 6.4)^1^	175 ± 10 (pore size: 6.4)	n.d.	+SEM
BNP (beer NP)	52.7 ± 20.7^1^	55.9 ± 29.5^1^	−7 ± 4	+FM
*Microparticles*				
MP-SiO	3012 ± 113	n.d.	−36.1 ± 2	

The average size of indicated nanomaterials was determined in the dry state (TEM) as well as in buffer-A by DLS. Zeta potentials were determined with a Zetasizer. Values are mean ± s.d. from three independent experiments. NP–bacteria interaction was verified by the indicated methods: *FM* (*quantitative) fluorescence microscopy, *EM* electron microscopy (SEM/TEM), *EDX* energy dispersive X-ray spectroscopy. Fluorescent labels (R = Rhodamine; G = FITC; B = AF350). SiP SIPERNAT^®^

**Fig. 1:**
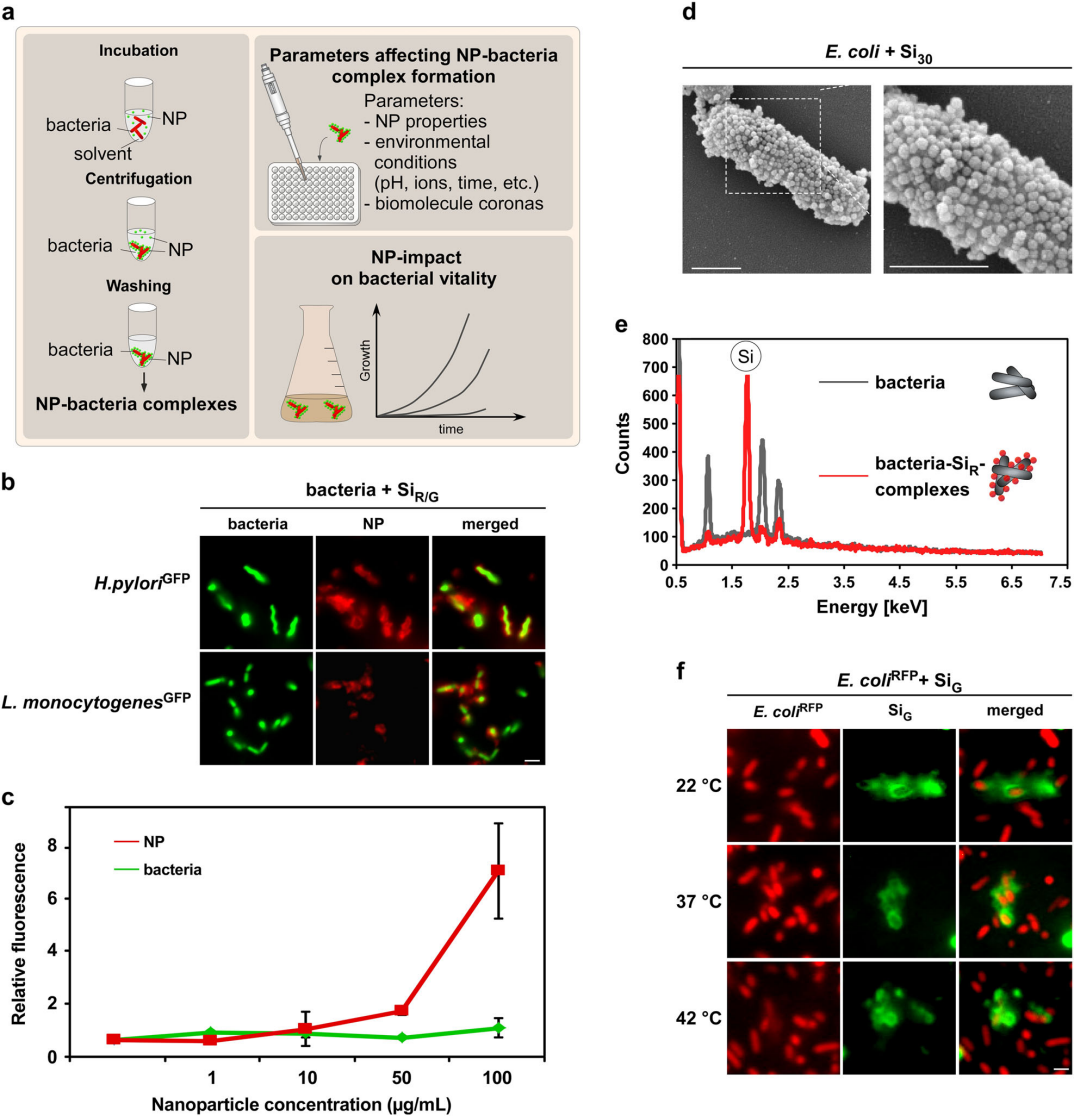
NPs rapidly and stably adsorb to *H. pylori* and other enteric pathogens. **a** Workflow to analyze material and environmental parameters affecting NP–pathogen interactions. Following co-incubation in media, such as PBS or simulated physiological fluids, NP–bacteria complexes can be harvested by mild centrifugation. Unattached pristine NPs remain in the supernatant and are removed. NP–bacteria complex formation can be analyzed via different methods after various time points under variable experimental conditions to investigate their impact on complex formation. **b** In situ complex formation of pristine NPs with autofluorescent pathogens. Indicated living bacteria were incubated with pristine fluorescent silica NPs (Si_R/G_) as shown in **a**, and analyzed by microscopy without fixation. Scale bar 2 µm. **c** Quantification of NP–bacteria interaction using the ArrayScanVTI automated microscopy platform. 1 × 10^6^ red fluorescent bacteria were incubated with the indicated concentrations of green fluorescent pristine NPs, and complexes analyzed in 96-well plates. A minimum of 1000 NP–bacteria complexes/well was analyzed for green and red fluorescence in triplicates using the TargetActivation assay. Increasing concentrations of NPs resulted in increased binding to bacteria. Red and green fluorescence intensity of complexes is displayed. As a control, the signal of GFP-expressing bacteria remains constant. **d** SEM visualizing assembly of pristine Si NP onto *E. coli*. Exposure: 10 min in PBS. Scale bars 1 µm. **e** Si NP detected on the surface of *H. pylori* by EDX. Elemental Si was absent on bacteria. **f** Variations in temperature (8–42 °C) during NP–bacteria incubation (5 min, PBS) did not affect NP-assembly. Complex formation was analyzed by live cell microscopy. Scale bars 2 µm. Images are representative of three independent experiments

We hypothesized that the effects of NPs on microbiota are strongly influenced by their physical contact and the nature of the interactions between the NPs and the microbes, as well as by how these physical phenomena are influenced by the dynamic physiological environments of the oral-GI uptake route. Due to a high number of uncontrollable variables, it is impossible to dissect these types of interactions in vivo, e.g., by analyzing digested, NP containing, food from the gut. As NP–bacteria interactions in physiological environments of the oral-GI uptake route occur in the liquid and not the dry interface, we developed a standard operating procedure, allowing us to study and quantify the kinetics of NP–bacteria complex formation under controllable experimental conditions, such as time, temperature, pH, or the concentration of ions and biomolecules (Fig. 1a, f, Supplementary Figs. S1 and S3).

**Fig. 2:**
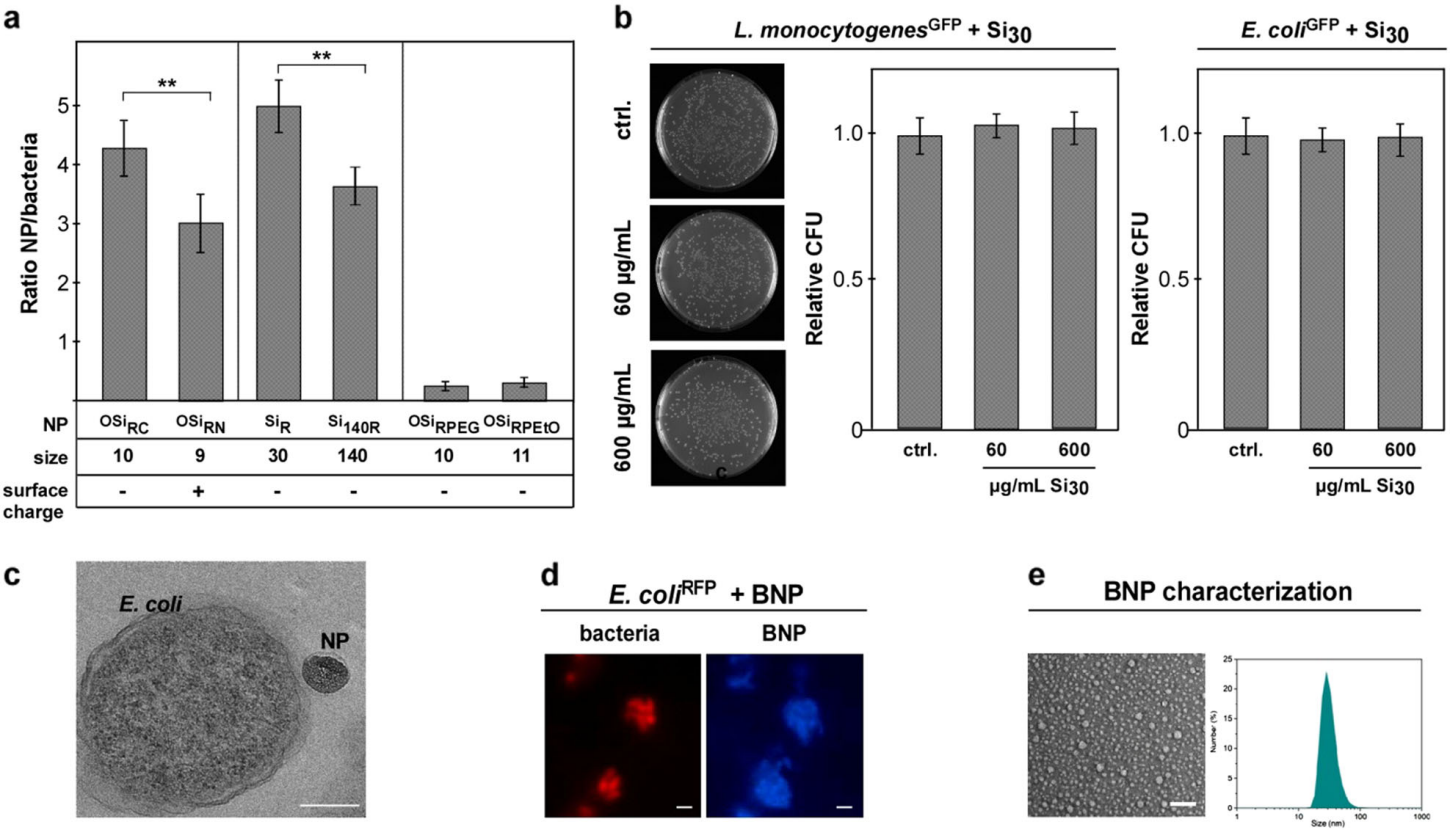
NPs’ physicochemical characteristics affect complex formation but not bacterial vitality. **a** NP size, charge, and stealth modification affect NP–*H*. *pylori* assembly. Quantification of NP (red)–*H. pylori* (green) interaction by automated microscopy. Reduced binding was observed for positively (OSi_RN_; ζ = +24 mV) versus negatively (OSi_RC_; ζ = −32mV) charged polymer NPs. Compared to small Si_R_ (∅ ~30 nm), larger silica Si_140R_ (∅ ~140 nm) displayed reduced binding. Stealth modification of polymer NPs (OSi_RPEG_/OSi_RPEtO_) reduced complex formation. Assays were performed in triplicates using pristine NPs. **b** Even high concentrations of pristine silica NPs (Si) did neither affect the vitality and growth of commensal microbes nor of tested enteric pathogens. CFU-assays of *L. monocytogenes*^GFP^ and *E. coli* 24 h after NP exposure are shown. **c** TEM demonstrating that exposure of *E. coli* to pristine Si NP did not result in bacterial cell wall damage or NP internalization. Exposure: Si_140_ (∅ ~140 nm) 600 µg/mL, 60 min in PBS. Scale bar 150 nm. **d** Autofluorescent NPs isolated from beer (BNP; blue) adsorb to *E. coli*^*mCh*^ (red). Left: Living bacteria were incubated with pristine BNP (∅ ~50 nm) for 10 min in PBS and analyzed by microscopy without fixation. Scale bar 2 µm. Right: SEM and DLS to determine BNP size distribution. Scale bar 150 nm

To mimic short- and long-term exposure scenarios, pathogens were exposed to NPs for various times and NP–bacteria complexes collected by mild centrifugation and washed, thereby removing any unbound NPs. Of note, we controlled that no free NPs were recovered by mild centrifugation (data not shown). To visualize NP–bacteria interactions in situ, we used various fluorescent silica NPs (Si NPs) in combination with transgenic models, producing red or green autofluorescent pathogens (Tables 1 and 2; Supplementary Fig. S1b). Fluorescence microscopy revealed a concentration-dependent rapid binding of NPs to enteric commensal bacteria and pathogens, including *H. pylori* and *Listeria monocytogenes* (Fig. 1b, c; Supplementary Fig. S1c). NP-binding was also found for so called “probiotic” bacteria, such as *Lactobacillus acidophilus*, *Bifidobacterium lactis*, and *Streptococcus thermophilus* species used in the fermentation of acidic milk products, such as yogurt (Supplementary Fig. S1d). Also, NP–bacteria complex formation was demonstrated by magnetic separation of the complexes using iron oxide NP contrast agents, which are normally used for GI-imaging (Supplementary Fig. S2a). Based on these results, we feel that centrifugal force may have only a minor impact on NP–bacteria complex formation. Complexation was confirmed by further independent methods, including scanning electron, transmission electron, and atomic force microscopy (SEM/TEM/AFM) as well as by energy-dispersive X-ray spectroscopy (EDX) (Tables 1 and 2; Fig. 1d–e; Supplementary Fig. S2b; Supplementary Table S1). SEM showed that the bacteria’s surface was coated with Si_30_ NPs at a level of about 60–80% coverage (Fig. 2d). In contrast, the NPs that tended to aggregate in physiological buffers, such as ZnO, preferentially adsorbed to the bacteria as NP clusters (Supplementary Fig. S2c). Kinetic analyses further demonstrated that the NP coating formed rapidly (<30 s) and was not affected by variations in temperature (4–55 °C) (Fig. 1f).

**Table 2:** Bacteria characterization

Bacteria	TEM diameter in dry state±s.d. (µm)	Gram	Zeta potential±s.d. (mV)	Disease relevance	NP binding
*Escherichia coli* ^GFP/−^	1.1 ± 0.2 × 2.3 ± 0.1	Negative	−74.2 ± 0.35	–	+FM/SEM/EDX
*Escherichia coli* ^RFP^	1.3 ± 0.3 × 2.6 ± 0.4	Negative	−72.1 ± 0.4	–	+FM/SEM/EDX
*Helicobacter pylori* ^GFP/−^	0.7 ± 0.1 × 2.9 ± .0.5	Negative	−6.6 ± 1.5	Gastritis, gastric cancer, ulceration, and MALT^53^	+FM
*Listeria monocytogenes* ^GFP/−^	0.4 ± 0.1 × 1.7 ± 0.3	Positive	−14.1 ± 1	Listeriosis, sepsis, and meningitis^54^	+FM
Enteropathogenic *E. coli*^GFP/−^ (EPEC)	0.6 ± 0.2 × 2.3 ± 0.3	Negative	−11.4 ± 1.1	Diarrhea and dyspepsia	+FM
*Shigella flexneri* ^GFP^	0.5 ± 0.1 × 2.2 ± 0.4	Negative	−22.2 ± 0.5	Dysentery, sepsis, and pneumonia	+FM
*Salmonella enterica* ^SL7207/*^	1.1 ± 0.5 × 3.4 ± 0.7	Negative	−16.6 ± 1.4	Enterocolitis and antitumoral effect^55^	+FM
*Lactobacillus acidophilus*	1.3 ± 0.2 × 8.1 ± 0.6	Positive	−38.2 ± 0.8	Probiotic and antimicrobial^56^	+FM
*Bifidobacterium lactis*	1.2 ± 0.3	Positive	−34.4 ± 0.6	Probiotic, antibacterial^57^	+FM
*Streptococcus thermophilus*	1.1 ± 0.2	Positive	−6.4 ± 0.2	Probiotic^58^	+FM

The average size of the different bacteria was determined in the dry state (TEM). Zeta potentials were determined with a Zetasizer. NP-binding was detected by the indicated methods (*FM* fluorescence microscopy, *SEM* scanning electron microscopy, *EDX* energy-dispersive X-ray spectroscopy). Values are mean ± s.d. from three independent experiments.

### NP–bacteria complex formation is affected by the NPs’ physicochemical characteristics

Although all types of tested NPs attached to bacteria, we noticed differences in binding depending on the NPs’ specific characteristics. As an example, we examined the behavior of Si NPs as a model for food-relevant NPs (Fig. 2a). Fluorescence-based automated quantification of complex formation revealed reduced binding for positively charged (OSi_RN_, ζ = +24 mV) vs. negatively charged (OSi_RC_, ζ = −32 mV) polymer NPs of similar size (Fig. 2a; Table 1; Supplementary Fig. S3a). Notably, all the bacteria studied had a net negative overall surface charge, and therefore one might have expected that negatively charged NPs would not bind due to electrostatic repulsion. However, anionic NPs, such as Si NPs, were found to bind efficiently to the surfaces of anionic bacteria (Fig. 2a; Tables 1 and 2). Also, less-negatively charged Si NPs (Si_N_R_, ζ = −8 mV) did not show significantly improved binding (Fig. 2a). Hence, assembly of NPs on bacteria cannot be simply predicted by the rules of colloidal electrostatics. NP–bacteria binding may have occurred for a number of reasons: (i) the electrostatic repulsion could be reduced by counter-ion screening effects, so that van der Waals attraction dominated and (ii) bacteria exhibit surface charge heterogeneity, thus positive (supra)molecular patches on their surfaces may primarily interact with the anionic NPs.^33^ Small (∅ ~30 nm) Si NPs bound more efficiently compared to larger ones (∅ ~140 nm) (Fig. 2a, Supplementary Fig. 3b), indicating that NP size is critical. Again, this effect may be due to surface heterogeneity effects on the bacteria surface—small NPs may be able to bind to small cationic patches on the bacteria surface, whereas large ones were not. Notably, surface modification with steric molecules, such as poly(ethylene glycol) (PEG) or poly(2-ethyl-2-oxazoline) (PEtO), applied in academia and industry to reduce overall protein binding (Supplementary Fig. S3a), efficiently reduced NP attachment to bacteria (Table 1; Fig. 2a; Supplementary Fig. S3a). This data suggests their use as chemical tools to rationally modulate NP–microbe complex formation. Of note, even exposure to high doses of those NPs that are typically used in the food industry did not affect the vitality and growth of commensal microbes or enteric pathogens when examined in liquid culture assays (Fig. 2b). Moreover, we did not observe that Si NPs were able to directly penetrate the rigid bacterial cell wall (Fig. 2c). Often, NP–bacteria interactions have been analyzed by electron microscopy only. Despite advantages concerning resolution and visualization of structural details, most EM techniques are low throughput and require harsh fixation and staining procedures, including chemical cross-linking, drying, and high vacuum. Such procedures can result in artifacts, such as membrane rupture of bacteria, leading to the impression that NPs can easily penetrate the surface of microbes.^16^ To further underline the relevance of our observation for food, we attempted to isolate NP from relevant consumer products. As NP isolation from solid or even (pre)digested food is rather complex, we focused on consumer-relevant liquid products. According to previous reports, the presence of carbon-based NPs seems to be generated by heating sugar solutions, as is also occurring during beer brewing. As beer is consumed worldwide, we isolated and purified naturally occurring carbon-based NPs from beer (BNP) via size exclusion chromatography (Supplementary information). The autofluorescent BNP were characterized by DLS, and ζ-potential measurements, fluorescence spectroscopy, and TEM (Table 1; Fig. 2d, e; Supplementary Fig. S2d). As shown in Fig. 2d, the blue fluorescent BNP also efficiently adsorbed to different enteric bacteria.

### Adsorption of NPs to bacteria reduces NP-mediated toxicity

**Fig. 3:**
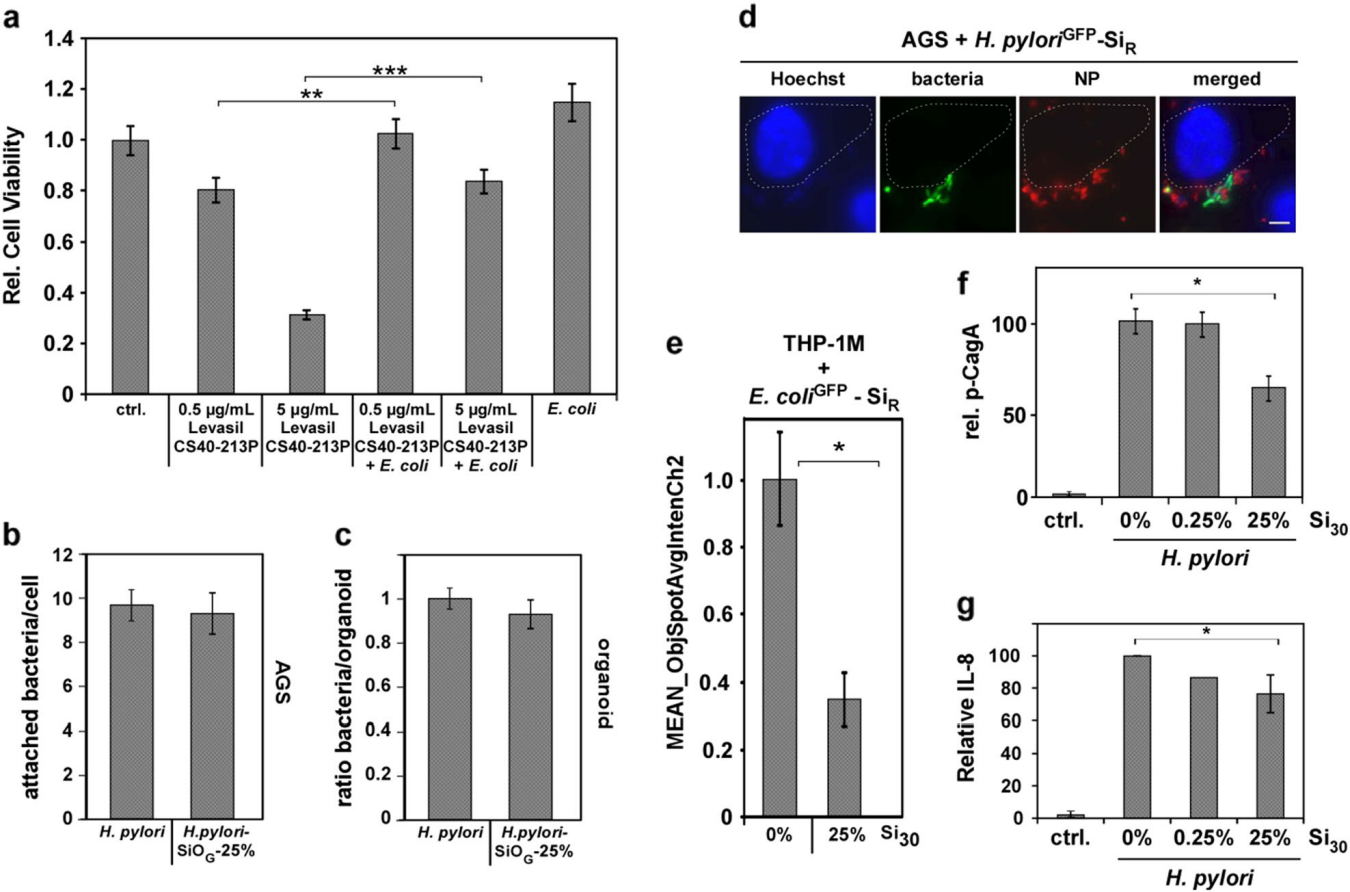
NP-binding impacts bacterial pathobiology and fate. **a** Adsorption of Levasil CS40-213P to bacteria reduced NP toxicity. 2 × 10^5^ human gastric epithelial (AGS) cells were either exposed to 0.5 or 5 µg CS40-213P or to 0.5 or 5 µg CS40-213P pre-incubated with 1 × 10^7^ bacteria for complex formation. Cell vitality was assessed after 6 h. **b** Live cell fluorescence microscopy visualizes attachment of NP–bacteria complexes. AGS cells were exposed to NP–*H. pylori*^GFP^ complexes and analyzed 16 h later. Scale bar 10 µm. **c** NP-coating does not affect cellular attachment of *H. pylori*. AGS cells were exposed for 90 min to Si_R_–bacteria complexes. Attachment was analyzed by confocal fluorescence microscopy. Assays were performed in triplicates, each with a minimum of 100 cells examined. NP–*H. pylori*^GFP^ complexes were prepared applying Si_30_ concentrations estimated to maximally cover approximately 25% of the bacterial surface (1 × 10^8^ bacteria, 600 µg/mL Si_30_; 10 min PBS). **d** 3D gastric organoids from normal human corpus mucosa were infected with pristine bacteria or Si_R_ NP–*H. pylori*^GFP^ complexes and analyzed by confocal fluorescence microscopy 8 h later. Bacteria and Si_R_–*H. pylori* complexes attached to 3D organoid structures equally well. NP–*H. pylori*^GFP^ 25% complexes: 1 × 10^8^ bacteria, 600 µg/mL Si_30_; 10 min PBS. **e** NP-coating reduces cellular uptake of bacteria into human THP-1M macrophages. Automated microscopy demonstrates reduced internalization of Si_R_–bacteria complexes. A minimum of 1000 cells was analyzed/well. Complexes 25%: 1 × 10^8^ *E. coli*, 600 µg/mL Si_30_, 10 min PBS. **f** Densitometric quantitation of phosphorylated CagA normalized to β-actin levels in all four experiments. Cells were infected with *H. pylori* or NP–*H. pylori*^GFP^ complexes. At 4 h post infection, CagA and phosphorylated CagA (p-CagA) were analyzed in cell lysates by specific antibodies. **g** Coating of *H. pylori* with silica NPs (Si_30_) results in a NP concentration-dependent decrease in IL-8 secretion. IL-8 was quantified by ELISA in AGS cell supernatants (*n* = 4). The amount of IL-8 in the sample *H. pylori* without NPs was set to 100%

Next, we studied the consequences of NP–bacteria association on the fate and (patho)biology of both the NPs and bacteria using cellular models. Toxic effects have been reported for various NPs, including silica-based or metal oxide NPs.^34,35^ Thus, we investigated the relevance of NP–bacteria complex formation on cytotoxicity. When human AGS gastric epithelial cells were exposed to silica NP–bacteria complexes in comparison to bacteria or NPs alone, we noticed a significant reduction of silica NP-induced toxicity for the complexes (Fig. 3a). Thus, NP binding to the bacteria surface seems to reduce the number of reactive sites on the NPs capable of interacting with epithelial cells in the GI. NP-binding to enteric pathogens did not affect cellular attachment but impacted pathogenic signaling.

As a controllable model to evaluate the impact of food-relevant NPs interacting with pathogens and cells in the gastric environment, we studied gastric cancer-associated *H. pylori*. Here, recognition and attachment to target cells is key for the subsequent infection and cellular reprogramming by *H. pylori*.^21^ When gastric epithelial AGS cells were infected with *H. pylori* or Si–*H. pylori* complexes, both were detectable at the cell membrane (Fig. 3b). Fluorescence-based quantification by confocal microscopy revealed that NP-coating did not significantly affect attachment of *H. pyloris* to the surface of AGS cells (Fig. 3c). Hence, mere mechanical coating of bacterial surfaces appears not to be sufficient to block initial steps in the infection cycle.

Although cell models are useful screening tools for providing mechanistic insights, 3D organoids more closely mimic the complex physiology and structure of human organs.^36,37^ Hence, although neglected so far, such systems should also be used to study processes at the nanobio interface, thereby reducing excessive animal experimentation. 3D gastric organoids were generated from normal human corpus mucosa cells and infected with pristine bacteria or Si NP–*H. pylori* complexes. Fluorescence-based quantification showed that Si NP–*H. pylori* complexes attached to 3D organoid structures to a similar extent as pristine bacteria (Fig. 3d, Supplementary Fig. S4).

Even though a NP-coating around the bacteria did not impact their cellular attachment, we hypothesized that this coating may influence their cellular uptake, as target or immune cells are primarily facing NP–bacteria hybrid structures rather than pristine bacteria. Differences in the surface characteristics of particles are known to impact their uptake by various cell types; thus we compared the cellular uptake of NP–bacteria complexes vs. bacteria. Quantification of uptake by high-throughput automated fluorescence microscopy revealed a significantly reduced internalization of NP-coated bacteria compared to bacteria alone for human epithelial cells as well as for macrophages (Fig. 3e). We subsequently examined the NPs’ impact on pathogenic signaling pathways. *H. pylori* attachment to host cells triggers the assembly of the type IV secretion system (T4SS) to inject CagA into cells.^21,38,39^ CagA phosphorylation as well as IL-8 induction can thus be used as reliable biomarkers for a functional T4SS and a manifested *H. pylori* infection, representing key steps in the development of inflammation-driven gastric cancer.^39^ To investigate the impact of a NP-coating in this context, gastric AGS cell lines were infected with NP–*H. pylori* complexes or bacteria. In order to avoid potential cellular artefacts induced by excessive NP doses, NP–*H. pylori* complexes were prepared by applying pristine Si NP concentrations estimated to maximally cover about 25 or 0.25% of the bacterial surface, respectively. Immunoblot-analysis of AGS cells infected with Si–NP *H. pylori* complexes revealed a concentration-dependent decrease of intracellular phosphorylated CagA (Fig. 3f). As an additional indicator for decreased *H. pylori* pathogenesis, a concentration-dependent decrease in IL-8 secretion was observed, when food-relevant silica NPs, such as Si_30_ and SiP were attached to bacterial cells prior to cell line infection (Fig. 3g). Our results demonstrate that assembly of food relevant, nonbactericidal NPs can indeed attenuate the pathobiological behavior of *H. pylori* and potentially of other enteric pathogens.

### Biomolecule coronas reduce NP–bacteria complex formation

**Fig. 4:**
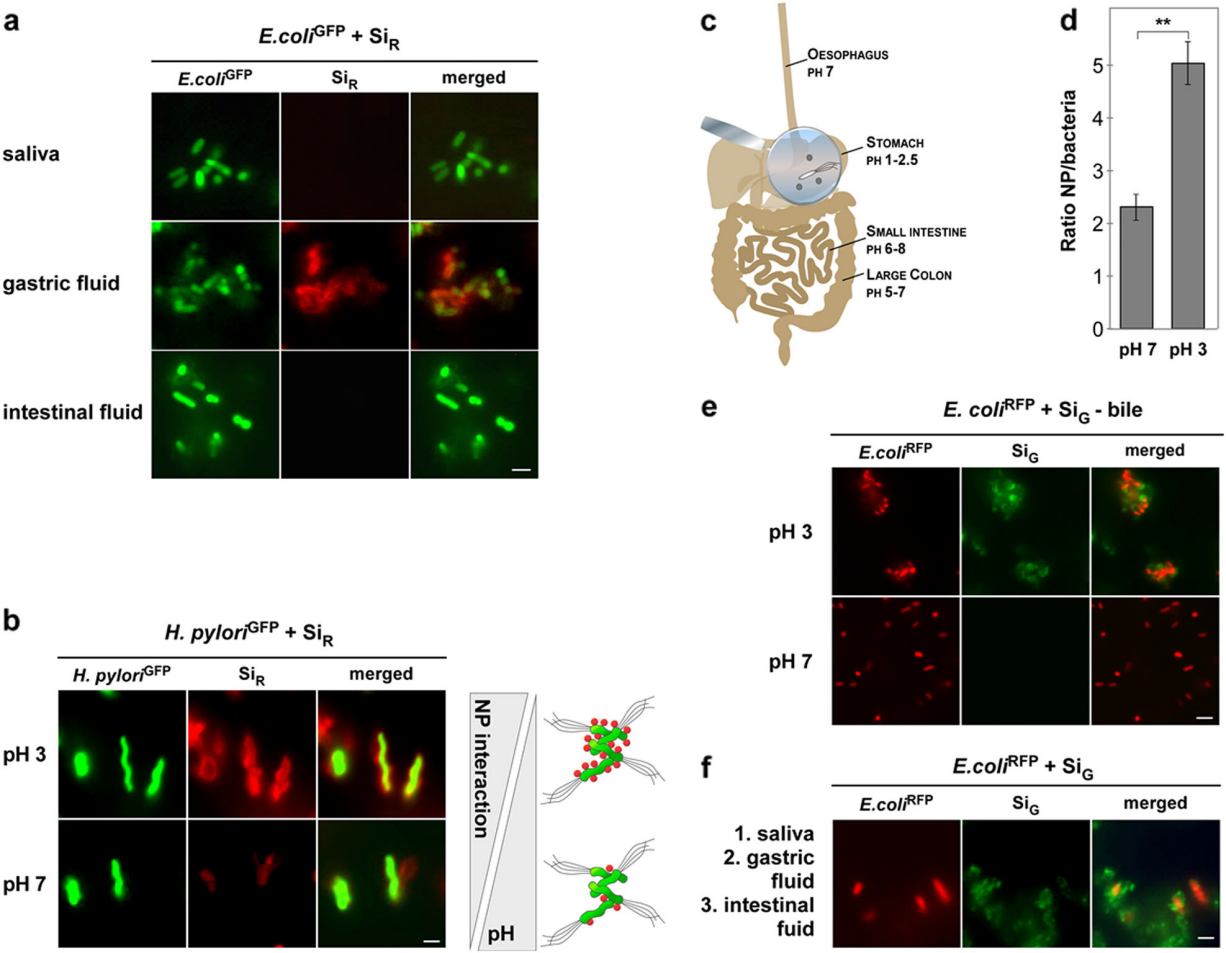
Environmental conditions of the oro-gastro-intestinal route affect NP–bacteria complex formation. **a** NP–bacteria complex formation is inhibited by biomolecule coronas forming in saliva or intestinal fluid, which could be overcome by incubation in acidic gastric fluid. **b** Acidic pH enhances NP adsorption to bacteria. *H. pylori* cells were incubated with Si_R_ at pH 7 in PBS or pH 3 in artificial gastric juice and analyzed by live cell microscopy. Corona-covered NP–bacteria complex formation increased with low pH. **c** Illustration of pH variations along the oro-gastro-intestinal route. **d** Quantification of pristine Si_R_ (red)—*H. pylori* (green) complex formation by automated microscopy at indicated pH. A minimum of 1000 NP–bacteria complexes/well was analyzed for green and red fluorescence using the TargetActivation assay. Columns show the mean ± s.d. from three independent experiments. Assays were performed in triplicates. **e** Exposure of NPs to intestinal bile extract (pH 7, lower panel) reduced complex formation with bacteria, which could be restored by acidic pH (pH 3, upper panel). **f** Sequential exposure of NPs and bacteria in the respective physiological fluids demonstrated that bacteria exposed to saliva still adsorbed to NPs in acidic environments, and complexes remained stably associated in intestinal fluid. Scale bars 2 µm. All images are representative of three independent experiments

After oral uptake, NPs as well as pathogens pass through various regions within the human oro-GI tract, which contain complex and quite diverse physiological fluids, including the mouth, stomach, small intestine, and colon.^3,4^ Each of these regions contains a mixture of various biomolecules that may adsorb to the surfaces of NPs, and therefore may alter their surface properties and GI fate. Besides proteins, mucins, sugars, phospholipids, bile salts, and mineral ions may contribute to forming a complex biocorona on the NP surface.^4,18^ It is, therefore, important to understand the influence of such complex molecular environments of the oro-GI tract on the fate of NPs and bacteria. In the mouth, both NPs and bacteria encounter saliva, which is a hypotonic fluid with low-ionic strength containing calcium, phosphate, carbonate, and thiocyanate ions.^40^ In addition, proteins such as MUC7, secretory IgA, and lactoferrin are present in saliva, constituting the salivary immune defense system that promotes the clearance of xenobiotics due to agglomeration effects.^40^ Binding of salivary proteins to NPs was confirmed by sodium dodecyl sulfate polyacrylamide gel electrophoresis analysis (Supplementary Fig. S5a). The formation of a GI tract-relevant biomolecular corona was further confirmed for artificial gastric and intestinal fluids, containing digestive enzymes and other proteins (Supplementary Fig. S5b,c). The impact of such biomolecular coronas on the interaction of NPs with bacteria was not investigated previously. Notably, all physiological biomolecular coronas concentration-dependently reduced NP–bacteria complex formation, except for the acidic artificial gastric fluid (Fig. 4a). Here, enhanced assembly of the NPs on *H. pylori* or other bacteria was observed, apparently capable of overriding the inhibitory impact of biomolecule coronas (Fig. 2a). Thus, we examined the effects of pH variations in physiological environments on NP–bacteria adsorption (pH 3–8). Notably, we found that NP–bacteria assembly was significantly enhanced in acidic environments, i.e., pH 3–5 (Fig. 4b–f). Also, exposure of pristine NPs to bile extract at neutral pH 7 reduced NP adsorption, which could be restored by lowering the pH (pH 3) (Fig. 4e). Subsequently, we simulated the physiological environments of oral uptake and GI-passage by sequential exposure of NPs and pathogens first to saliva, then to gastric fluid, and finally to intestinal fluid. After each incubation, a washing step was performed to separate bacteria from unbound NPs. Here, we found that bacteria exposed to saliva were still able to bind NPs in acidic environments, underlining again the relevance of the GI tract for bacteria–NP interactions (Fig. 4a, f).

## Discussion

Food-grade NPs are increasingly being utilized as functional ingredients in the food industry, and so it is important to understand how they behave in and potentially affect the complex environment of the human oro-GI tract. In this study, we focused on the interactions of model as well as food-relevant NPs with both beneficial (probiotic) and detrimental (pathogenic) bacteria under simulated GI conditions. In particular, we demonstrated that various types of NPs, representative of the NPs currently or potentially used in the food sector as well as naturally occurring “hard” NPs, form stable complexes with both commensal microbes and enteric pathogens. A range of complementary analytical techniques showed that NP size was the most relevant determinant of NP–bacteria complexation, rather than core material type or surface charge. Our results also highlighted that binding efficiencies cannot be predicted based on colloidal electrostatics, as negatively charged NPs bound to negatively charged bacterial surfaces of both Gram-positive and Gram-negative bacteria. Indeed, small negatively charged NPs of different materials formed complexes with bacteria more efficiently than larger positively charged ones. Nevertheless, NP–bacteria complexation could be chemically prevented by coating the NPs with a polymeric layer that generated strong steric repulsion. Our study further demonstrated that low pH, such as that characteristic of the gastric environment, significantly enhanced NP–bacteria complexation and even overrides the inhibitory effect of physiological biomolecule coronas.

These findings may stimulate the development of more effective NP-based systems for the treatment of GI infections, since the interaction of NPs with *H. pylori* reduced the severity of infection in our gastric model. Additionally, environmental factors, such as dietary components and micronutrients as well as the GI microbiota, seem to affect the balance between *H. pylori’s* role as a commensal or a pathogen.^3,27,32^ We showed that NPs naturally present in food as well as synthetic nanosized food additives may impact the bacterial life cycle. Hence, one may speculate that during evolution not only soluble chemicals but also (nano)particulates may have contributed to shaping the microbiome as well as its interaction with the human host. It is now accepted that human cells respond not only to soluble molecules but also to mechanical forces as induced by particulates of various sizes.^3,4^ Although still speculative, one may envisage to rationally exploit natural or synthetic nanosized food additives in the future, to achieve positive “side-effects” by shaping the microbiome and/or by inhibiting enteric pathogens, such as *H. pylori*. Dissecting and understanding the underlying molecular and physicochemical mechanisms of NP–bacteria crosstalk will also be important to define parameters regulating the addition of nanosized additives to foods as a “safe by design” strategy for consumers’ health.

## Methods

### Chemicals

Bulk chemicals and reagents were purchased from Sigma Aldrich, Gibco, and Invitrogen.

### Nanoparticles

Silica NPs of different size, fluorescence, and surface modifications were purchased from Kisker Biotech. Polymer, metal oxide, and carbon NPs as well as microparticles were synthesized or are commercially available (Sigma Aldrich; MainzScreeningCenterUG). All NPs were characterized regarding average size and zeta potential by TEM, SEM, AFM, DLS, and zeta potential measurements as reported elsewhere^3,41,42^ For further details see supplementary information.

### Isolation of beer nanoparticles (BNP)

To isolate BNP, 200 mL of a common pilsener brew were evaporated to dryness, resuspended in 10 mL water, and filtered through a syringe filter (220 nm). BNP were further purified using size exclusion chromatography (Sephadex G-25) and fractions identified by a UV-lamp (405 nm).

### Bacteria cultivation

Bacteria strains, genetically modified to express red fluorescent protein tdTomato or green fluorescent protein GFP, were used for fluorescence microscopy analyses. Cultures were grown in the respective media at 37 °C and 140 rpm overnight as described.^26^ For details see supplementary information.

### NP–bacteria complex analyses

To analyze NP–bacteria complex formation, NPs and bacteria were incubated in varying media, at different temperatures, and for several time points, as indicated. NP–bacteria complexes were harvested under mild centrifugation conditions (10 min, 3000 rpm, 20 °C), washed twice (phosphate-buffered saline (PBS)), and used for experiments at specific counts. Different independent methods, including automated fluorescence microscopy, SEM/TEM, or EDX were applied to analyze binding of NPs to bacteria. For further details see supplementary information.

### CFU quantification

Colony-forming units (CFU) were determined to assess NPs’ impact on the vitality of bacteria. Bacteria were incubated with NPs and washed with PBS. Different dilutions were plated on solid LB media plates as described.^12,26^ Colonies were counted after incubation of plates at 37 °C for 24 h.

### Cell culture

Cell lines, namely colorectal epithelial (Caco2), gastric cancer (AGS, MKN-28), and human monocytic leukemia (THP-1) cell lines, were maintained, and authenticated as described previously (for details see supplementary information).^43–46^ In short, they were passaged every 2–3 days or as appropriate and used for a maximum of 20 passages. Cell vitality was assessed as described.^47,48^ Cytokine profiles were obtained using the commercially available human IL-8 ELISA test kit (Biolegend).

### Microscopy

Confocal laser scanning, fluorescence microscopy as well as automated high content microscopy were applied to visualize or quantify NP–bacteria complex formation. Moreover, uptake and cellular localization of fluorescent bacteria, NPs, and NP–bacteria complexes were analyzed using unfixed samples as described.^42,49–51^ The ArrayScanVTI automated microscopy platform was used to quantify NP–bacteria interaction. In short, 1 × 10^6^ green fluorescent bacteria were incubated with red fluorescent NPs. A minimum of 1000 NP–bacteria complexes were analyzed regarding the fluorescence signal per well with the TargetActivation assay. For further details see supplementary information.

### Gastric model

Human gastric organoids were cultured as previously described.^52^ Organoids consisting of approximately 4000 cells were microinjected 10 days after seeding with green fluorescent *H. pylori* or complexes of red fluorescent silica NPs and green fluorescent *H. pylori* cells at a multiplicity of infection of 50.^52^ For further details see supplementary information.

### Statistical analysis

Statistical significance was determined by using the Mann–Whitney test or paired *t* test assuming significance at ^*^P = 0.05; ^**^P = 0.01; ^***^P = 0.005 as described previously.^43^

## Electronic supplementary material

Supplemental Information

## Data Availability

The authors can confirm that all relevant data are included in the paper and/or its supplementary information files.

## Electronic supplementary material

**Supplementary information** accompanies the paper on the *npj Science of Food* website (10.1038/s41538-018-0030-8).
