# Supplementary material for: Nanosized food additives impact beneficial and pathogenic bacteria in the human gut: a simulated gastrointestinal study
Source: NPJ Sci Food. 2018 Dec 4;2:22. doi: 10.1038/s41538-018-0030-8 (PMC6420113; doi:10.1038/s41538-018-0030-8)
Supplement: Supplementary file 1 — Supplemental Information [file 41538_2018_30_MOESM1_ESM.pdf]

SUPPLEMENTARY INFORMATION

Nanosized food additives impact beneficial and pathogenic bacteria in the human gut: A simulated gastrointestinal study

Svenja Siemer[1], Angelina Hahlbrock[1], Cecilia Vallet[2], David Julian McClements[3], Jan Balszuweit[4], Jens Voskuhl[4], Dominic Docter[1], Silja Wessler[5], Shirley K. Knauer[2], Dana Westmeier[1] , and Roland H. Stauber[1,]*

[*]Corresponding author: Roland H. Stauber, roland.stauber@unimedizin-mainz.de

Table of Contents

SUPPLEMENTARY METHODS *page*

Nanoparticle synthesis and characterization. S2
Bacterial cultivation. S3
NP-bacteria complex formation and characterization. S3
Protein separation by 1-D gel electrophoresis. S4
Cells, cell culture and toxicity assays. S4
Immunoblotting. S5
Fluorescence microscopy. S5
Scanning electron microscopy (SEM). S5
Transmission electron microscopy (TEM) of NP-bacteria complexes. S6
Atomic force microscopy (AFM). S6
Energy dispersive X-Ray spectroscopy (EDX). S7
Quantification of bacteria internalization in human cells by automated microscopy. S7
PCR. S8
Human Tissue Material. S8
Organoid culture and infection. S8
Reagents. S9
Statistical Analysis. S9

SUPPLEMENTARY TABLES

S1 EDX analysis. S10
S2 Gastric fluid simulation media. S10

SUPPLEMENTARY FIGURES

S1 Investigation of NP-bacteria complex formation. S11
S2 NPs rapidly and stably adsorb to various pathogens under physiologically relevant conditions. S12
S3 NPs' physico-chemical characteristics determine binding to bacterial surface. S13
S4 NP-bacteria complexes attached to 3D organoid structures. S14
S3 SDS-PAGE demonstrates that all tested NPs acquire a protein corona. S15

SUPPLEMENTARY REFERENCES S16

SUPPLEMENTARY METHODS

Nanoparticle synthesis and characterization. Blue (AF350), red (rhodamine B) or green (FITC) fluorescent as well as non-fluorescent amorphous NPs (Si) of different sizes, modified by different surface-functionalization (amine, carboxylate, and unmodified) were purchased from Kisker Biotech (Steinfurt, Germany) and used as described[1-2]. ZnO NPs were provided by the *DENANA* research consortium and prepared by flame spray pyrolysis as described[3]. Carbon black (CAS#1333-86-4) was purchased from Sigma Aldrich (Darmstadt, Germany). Multi-walled carbon nanotubes (CNT) reference materials were provided by the EU *Joint Research Center* Ispra, magnetic fluorescent iron oxide (Fe_2O_3) NPs were provided by the *NanoBEL* research consortium. Non-coated positively and negatively charged fluorescent (rhodamine B) poly(organosiloxane) NPs (POSi) modified by different surface-functionalization, such as poly(ethylene glycol) (PEG) or poly(2-ethyl-2-oxazoline), were synthesized and used as described[4]. Briefly, carboxy-functionalized NPs ($POSi_{RC}$) were obtained by polycondensation of different alkoxysilanes in aqueous dispersion. Positively charged NPs ($POSi_{RN}$) were synthesized by coupling of *N*,*N*-diethylethylethylendiamine. PEGylated NPs ($POSi_{RPEG}$) were prepared by adding PEG to maleimido-functionalized NPs. For synthesis of poly(2-ethyl-2-oxazoline)-modified NPs ($POSi_{RPEtO}$), poly(2-ethyl-2-oxazoline) was reacted with maleimido-functionalized NPs[4]. Sipernat® NPs (SiP) were provided by Hch. Hisgen GmbH & Co. KG. (Frankfurt a.M., Germany). Levasil CS40-213P and Levasil CS50-34P were bought from Obermeier GmbH & Co.KG. (Bad Berleburg, Germany) Silver and copper NPs were commercially available from Merck KgaA (Darmstadt, Germany). Endorem® was sourced from Guerbet (Villepinte, France). NPs were characterized by TEM, SEM, AFM, DLS, and zeta potential measurements as reported[1-2]. Briefly, transmission electron microscopy (TEM) imaging was performed using a Philips EM420 on carbon coated copper grids. The size and zeta potential (were determined with a Malvern Zetasizer NanoZS as described[5]. Average particle size as well as size distribution was determined by dynamic light scattering (DLS) using a Nicomp380 Submicrometer Particle Sizer (Nicomp Particle Sizing Systems) at 20°C operating at a scattering angle of 90°. DLS results are reported as the average of at least 3 runs, each containing 100 individual measurements.

Bacterial cultivation. For fluorescence microscopy experiments, genetically modified bacteria strains were used expressing red fluorescent protein tdTomato from the vector $^{(p)}$*gpdA::dTomato::his2A*$^{(t)}$ <ptrA> (here *E. coli*^{RFP}) or expressing green fluorescent protein GFP from the vector $^{(p)}$*gpdA::EGFP::his2A*$^{(t)}$ <ptrA> (here *E. coli*^{GFP}, *H. pylori*^{GFP}, *L. monocytogenes*^{GFP}). *E.coli*$^{GFP/RFP}$ were grown in LB medium with 100 µg/mL Ampicillin. *H. pylori* strain P12 (wild type; expressing Western CagA EPIYA-ABCC) has been described previously[6]. *H. pylori*^{GFP} and *L. monocytogenes*^{GFP} were cultured in BHI medium (37 g/L brain heart infusion) with 6 µg/mL Chloramphenicol and 10 g/L Cyclodextrin for *H. pylori*^{GFP} under microaerophilic conditions and with 30 µg/mL Chloramphenicol for *L. monocytogenes*^{GFP}.

NP-bacteria complex formation and characterization. Bacteria concentrations were determined using the Cell Counter Casy I-TT (Innovatis AG). Bacteria were washed five times before incubation with NPs under varying conditions in respect to media type, pH condition and temperature for varying time spans. NP-bacteria complexes were then harvested by mild centrifugation (10 min, 3,000 rpm, 20 °C), the pellet was resuspended in PBS buffer and another washing step (10 min, 3,000 rpm, 20°C) was performed before resuspension in PBS buffer. NP-bacteria complex formation was performed based on the calculation of theoretical NP-bacteria binding. The surface area of a single bacterium is ~13.5 µm^2 (assuming an ideal cylinder shape with hemispheres at both ends). The maximum area occupied by single NPs (Si_{30}; ∅~30 nm) corresponds to the cross-sectional area of ~706.9 nm^2. Hence, it is estimated that maximally ~2x10^4 NP (Si_{30}; ∅~30 nm) can bind to a single bacterium, not taking into account any possible uptake by the bacterial cell. Interaction studies under different pH conditions were performed in: NaOAc (1 M, pH 3 and 1 M, pH 5) and Tris/HCl (1 M, pH 8). Gastric fluid simulation media was prepared according to Supplementary Table S2. For the use in cell culture experiments, an amount of NPs corresponding to a theoretical coverage of 0.25 % or 25 % of the bacterial cell surface was used for the formation of complexes. As cells were exposed at a multiplicity of infection (MOI) of ~5, the maximum Si_{30} NP dose/cell can be calculated to be approximately 2,500 ng, assuming that all bacteria cells are covered with NPs as calculated and are coming into direct contact with the cell. In contrast, SEM data showed that even incubation with the amount of NPs corresponding to a theoretical coverage of 100 % of the cell surface resulted in a surface coverage of only ~60-80%. Thus, we assume that a maximum of ~1,500 Si_{30} NP are attached per bacterium.

Protein separation by 1-D gel electrophoresis. For 1-D gel electrophoresis, *E. coli*^{GFP} were incubated with magnetic FeO_G nanoparticles as described above. To subsequently separate the magnetic FeO_G NPs with bound proteins from the suspension, they were extracted from the biological media using a MidiMACS Separator (Miltenyi Biotec)[7]. Recovered FeO_G were then centrifuged and washed as described before[1]. Proteins were eluted from the recovered particles by adding 100 µL of SDS sample buffer (62.5 mM Tris-HCl pH 6.8; 2 % w/v SDS, 10 % glycerol, 50 mM DTT, 0.01 % w/v bromophenol blue) to the pellet and incubating at 95 °C for 5 min. The samples were separated on a 12 % SDS-polyacrylamide gel (Mini-PROTEAN®, Bio-Rad). Gels were run at the constant voltage of 200 V for 35 min, and stained with Coomassie brilliant blue R-250 (Bio-Rad). Protein quantification was performed using the BioRad Protein Assay (Bio-Rad) as reported[2].

Cells, cell culture, and toxicity assays. Authenticated and characterized cell lines were purchased from the American Type Culture Collection (ATCC), stocks prepared at early passages, and frozen stocks kept in liquid nitrogen. Thawed cells were expanded, routinely monitored by visual inspection as well as growth-curve analyzes to keep track of cell-doubling times, and were used for a maximum of 20 passages. Depending on passage number from purchase, cell line authentication was further performed at reasonable intervals by short tandem repeat (STR) profiling. Caco-2 (ATCC HTB-37), AGS (ATCC CRL-1739) and MKN-28 (CVCL-1416) cells were maintained in RPMI1640 medium (Sigma Aldrich) supplemented with 10% fetal calf serum (FCS, Biowest), L-glutamine (2 mM; Biowest), and Pen/Strep (100 U/100 µg/mL, Invitrogen) in a humidified CO_2 atmosphere (5 %) at 37°C as described[8]. THP-1 cells (ATTC TIB-202) were cultivated likewise, but in suspension and with addition of 20 % FCS. Differentiation of THP-1 cells towards macrophage-like cells (THP-1M) was triggered by the addition of PMA (50 ng/mL) for 24 h, followed by a washing step with PBS buffer and the addition of complete media over 48 h post differentiation. For infection experiments, cells were grown in culture dishes to a confluency of approx. 70%, counted, and then seeded into 35 mm glass bottom dishes (MatTek), 24- or 96 well-plates (Greiner Bio-One) at a concentration of 10^6 cells/ml. They were incubated for 24 h. One hour before infection, medium was replaced with serum free medium (RPMI 1640, 2 mM L-glutamine). Subsequently, cells were exposed to the respective bacteria suspension (+/- NPs) under standard cell culture conditions. Afterwards, cells were washed three times with PBS buffer to remove the largest part of unattached and non-phagocytosed bacteria. Infected cells were analyzed by different methods of

microscopy and PCR. Furthermore, the viability was measured using the CellTiter-Glo® Luminescent Cell Viability Assay (Promega). IL-8 in culture supernatants was measured using human IL-8 ELISA (Biolegend) according to manufacturer's instructions.

Immunoblotting. For bacteria NP interaction studies, immunoblotting was carried out as described previously[2]. For detecting *H. pylori* induced CagA phosphorylation, AGS cells were infected with *H. pylori*^{GFP} at a MOI of 100 as described before. After 4 hours of infection, cells were washed twice in PBS, harvested in lysis buffer (20 mM Tris pH 7.5, 100 mM NaCl, 1 % Triton X-100, 0.5 % DOC, 0.1 % SDS, 0.5 % NP-40) and analyzed by immunoblot as described[8]. Briefly, equal amounts of protein were separated by SDS-Page, transferred to nitrocellulose membranes and immunoprobed for phospho-tyrosine (α-pY99, Santa Cruz), total CagA (polyclonal rabbit α-CagA serum, Santa Cruz) or β-actin (α-β-actin, Sigma-Aldrich). Membranes were analyzed with the Odyssey Fc Imaging System (Li-COR Biosciences) using IRdye® 800CW α-mouse-IgG or IRdye® 700CW α-rabbit-IgG (Li-COR Biosciences). Band intensities were quantified using ImageStudio (Li-COR Biosciences) and intracellular p-CagA was normalized for β-actin.

Fluorescence microscopy. Bacteria-nanoparticle interactions and cellular internalization or attachment of bacteria were analyzed by using the AxioVert 200M microscope (Carl Zeiss). After the incubation of bacteria and nanoparticles, and washing as described, bacteria were directly given into 35 mm glass bottom dishes (MatTek) for the microscopy of NP-interaction. For the analysis of cellular infections, bacteria-infected cells were fixed with 4 % PFA for 10 min. Cell nuclei were stained with 0.5 µg/mL Hoechst 33342 in PBS for 30 min (excitation: 405 nm; emission: 430-480 nm). Image analysis and presentation were performed as described in detail elsewhere, using the Axiovision software (Carl Zeiss)[2].

Scanning electron microscopy (SEM). All samples were fixed with 2.5 % glutaraldehyde in phosphate buffer 0.1 M under vacuum for 24 h. Bacteria or NP-bacteria complexes were fixed on glass coverslips and examined using a field emission scanning electron microscope (Quanta 200 FEG) as described[9]. To avoid any charging of the samples, images were taken under low vacuum condition (0.9 mbar) with a large field gaseous secondary electron detection system. Different magnification levels (10,000x, 50,000x, 100,000x) were used. Beam energy (20 keV) and electron current density (Spot

3) were kept constant for all analyses. Scanning speed and integration time were selected to avoid beam damages to the bacteria substructures. Image analysis and presentation were performed using the FEI Eindhoven image software[9] (FEI Eindhoven).

Transmission electron microscopy (TEM) of NP-bacteria complexes. $1x10^6$ NP-bacteria complexes were resuspended in 500 µL of glutaraldehyde (2.5% in 100 mM phosphate buffer (PB), pH 7.2) and incubated for 2 h at room temperature (RT). After 3 washing steps (300 µL), osmification with 1% osmiumtetroxide in PB was conducted for 60 min on a rocker at 4 °C in the dark, followed by washing in pure PB (3x 20 min), again rocking at 4 °C in the dark. Then the bacteria were incubated in 30% and 50% aqueous ethanol solutions for 30 min each and were subsequently transferred into a 70% ethanol solution containing uranyl acetate (1 %) for 60 min at RT in the dark. Subsequently, 80%, 90% and 96% ethanol solutions (30 min each) and finally pure ethanol (2 x 20 min) were applied to entirely dry the specimens. To prepare the resin embedding, bacteria were first incubated in a 1:1 mixture of 100% ethanol and propylene oxide (PO; 30 min, RT), followed by 2 x 20 min in pure PO. For EPON® resin embedding the bacteria were processed through a series of PO:EPON® solutions (ratios PO:EPON® = 3:1, 1:1, 1:3; 45 min each) and finally in pure EPON® overnight at room temperature. To concentrate bacteria, samples were centrifuged for 8 min at 13,000 rpm/RT before they were transferred to 60 °C for 48 h for EPON® solidification. After trimming (Pyramitome®,Model 11800, LKB), solid EPON® blocs were cut on an ultramicrotome (Ultracut® E, Reichert-Jung) set to a thickness of 50 nm. Sections were then mounted on 200 mesh hexagonal copper grids and contrasted first with 1% uranyl acetate followed by 0.4% lead citrate for 3 min, each. TEM was then conducted on a LaB6-equipped Jeol 1400Plus (Akishima) using 120 kV. Images were recorded with a 4096x4096 pixels CMOS camera (TemCam-F416, TVIPS).

AFM. *E. coli* XL2-Blue™ were stained with Bac Light™ Red bacterial stain (Life Technologies) according to manufacturer's protocol. *E.coli* were washed three times with ddH_2O prior to paraformaldehyde fixation for 2 h at RT. After three additional washing steps, the suspension was diluted 1:10 in ddH_2O and 100 µl were transferred onto a microscopy slide which was left to dry over night at RT. For atomic-force microscopy experiments, a JPK instruments BioMAT Workstation was used combining fluorescence microscopy with atomic force microscopy (AFM). The epifluorescence microscope (EFM)

consisted of a Zeiss AxioImager.M2.m equipped with a HBO 100 mercury vapor lamp and a 100x air-objective (Zeiss EC Epiplan-NEOFLUAR 100x/0.9 Pol). Images were taken with a digital microscope camera (Zeiss, AxioCam MRm) and processed using Zeiss Zen 2 pro. A JPK instruments NanoWizard II and images were processed using the JPK SPM Image processing (v.3.x). To combine EFM with AFM, a shuttle stage was applied. The shuttle stage allows an examination of the same spot with both techniques with a spatial deviation of less than 5 µm[12]. Samples were visualized in intermittent contact mode with a line rate of 0.06 Hz and a tip velocity of 0.5 µm/s using cantilever CSC37, the force kept constant at 0.35 N/m (Mikromasch).

Energy Dispersive X-Ray Spectroscopy (EDX). Bacterial samples were fixed with 2.5 % glutaraldehyde. Bacterial surfaces were analyzed by EDX using an INCA 350 energy spectrometer (Oxford Instrument GmbH) equipped with a PENTAFET Plus Si(Li)-Platinum detection system directly coupled to the Quanta 200 FEG. Spectra were obtained from highlighted locations with the “Point&ID” mode of the INCA navigation software[9]. A minimum of three spectra were taken in the middle of the SEM images with an acquisition time of 60 s each and evaluated with INCA Energy software packages.

Quantification of bacteria internalization in human cells by automated microscopy. To quantify the internalization of bacteria into human cells, the automated ArrayScanVTI imaging platform (Thermo Fisher Scientific Inc.) was used as described previously[2]. Cells were seeded with an electronic multichannel pipette (Eppendorf) into 24-well cell culture plates (Greiner Bio-One) and incubated in a 5% CO_2 humidified atmosphere at 37 °C. Cells were infected with different bacteria suspensions (+/- NPs) for 90 min under standard cell culture conditions. After washing with PBS buffer for three times, cells were fixed with 4% PFA for 10 min and the nuclei were stained with 0.5 µg/mL Hoechst 33342 (excitation: 405 nm; emission: 430-480 nm). Images were analyzed using the SpotDetection assay. Briefly, for every cell, a binary image mask was created from the Hoechst staining signal (XF100 Hoechst 355/465) to define the region of interest. In the second channel (XF93 GFP 475/515), this circular mask was dilated (two pixels) to cover the whole cell and the spot number of the fluorescent bacteria was quantified within this mask. Scans were performed sequentially with settings to give sub-saturating fluorescence intensity. A minimum of 1,500 cells/well was recorded. PBS- and medium-

treated cells served as a negative control to correct for background fluorescence. Each experiment was performed in triplicates.

PCR. DNA of infected blood was isolated using the DNeasy Blood and Tissue Kit (Qiagen). The DNA concentration was measured with the Nanodrop-2000 (Thermo Fisher Scientific). PCR analysis was performed using the Phusion Blood Direct PCR Kit (Thermo Scientific). A typical PCR sample consisted of a 25-µL volume containing 0.5 µM of the primers (pGEX-GFP-for 5'- AAA CGA GGA GCT GTT CAC CGG -3'; pGEX- GFP-rev 5'-TTT CCA TGC CGT GAG TGA TGC C -3'), 1X Phusion Blood PCR buffer, 1X Phusion Blood II DNA Polymerase and 200 ng DNA of the respective sample. The following PCR conditions were applied: 5 min, 98 °C initial denaturation; 1 s, 95 °C cyclic denaturation; 30 s, 72 °C cyclic extension for a total of 35 cycles, followed by 1 min, 72 °C final extension (protocol provided by Thermo Scientific). PCR amplification products were mixed with 1X DNA loading dye (xylene cyanol) and separated on a 1% agarose gel with $0.3x10^{-3}$% ethidium bromide. Gels were run at the constant voltage of 75 V for 60 min, and the separation was documented with the transilluminator Multigenius Bioimagine (Syngene) at 260 nm.

Human Tissue Material. Human corpus tissue was obtained from 17 patients (12 men, 5 women; age range, 41–87 y) who underwent partial or total gastrectomy at the University Medical Centre Utrecht. Ten patients were diagnosed with gastric cancer and 7 patients were diagnosed with esophageal cancer. This study was approved by the ethical committee of the University Medical Centre Utrecht. Samples were obtained with informed consent.[10]

Organoid culture and infection. Glands were extracted from 1 cm^2 of human tissue using EDTA in cold chelation buffer[11] seeded in Matrigel (BD Biosciences), and overlaid with DMEM/F12 medium (Gibco) supplemented with penicillin/streptomycin (100 U), HEPES (10 mmol/L), GlutaMAX (1 % (v/v)), B27 (1 mM) (all from Invitrogen), and N-acetylcysteine (1 mmol/L, Sigma-Aldrich). Growth factors were added to the basal medium as indicated elsewhere[10]. The final human stomach culture medium (advanced DMEM/F12) contained the following essential components: 50 ng/mL epidermal growth factor (EGF) (Invitrogen), 10% noggin-conditioned medium, 10% R-spondin1–conditioned medium, 50% Wnt-conditioned medium, 200 ng/mL fibroblast growth factor (FGF)10

(Peprotech), 1 nmol/L gastrin (Tocris), and 2 mmol/L transforming growth factor (TGF)bi (A-83-01; Tocris). The facultative component was 10 mmol/L nicotinamide (Sigma-Aldrich). After seeding, 10 mmol/L rho-associated coiled coil forming protein serine/threonine kinase (RHOK) (Y-27632; Sigma-Aldrich) was added. Additional tested components were as follows: 100 ng/mL insulin-like growth factor (IGF) (Peprotech), 10 mmol/L p38 inhibitor (SB202190; Sigma-Aldrich), 3 mmol/L glykogensynthase-Kinase (GSK)3b inhibitor (CHIR99021; Axon Medchem), and 500 nmol/L prostaglandin E (PGE)2 (Tocris). Approximately 1 cm^2 of cancer tissue was cut into small fragments and washed in cold chelation buffer until the supernatant was clear. Fragments were subjected to enzymatic digestion by 1.5 mg/mL collagenase (Gibco) and 20 mg/mL hyaluronidase (Sigma-Aldrich) in 10 mL advanced DMEM/F12, supplemented with antibiotics (Primocin; Invivogen), for 1 hour at 37 °C while shaking. Cells were washed twice in advanced DMEM/F12, seeded into Matrigel, and overlayed with medium containing HEPES, GlutaMAX, penicillin, streptomycin, B27, n-acetylcysteine, EGF, Rspondin1, noggin, Wnt, FGF10, gastrin, TGFb inhibitor, and RHOK inhibitor as described[10].

Organoids were microinjected on day 10 after seeding with a MOI of approximately 50 unless otherwise stated. For calculation of MOI, organoids were disrupted into single cells by EDTA and cells were counted, resulting in approximately 4000 cells/organoid. To achieve a final MOI of 50, pristine *H. pylori*^{GFP} and *H. pylori*^{GFP} pre-incubated with NPs as described earlier were suspended in advanced DMEM/F12 at a density of $1x10^9$/mL and organoids were injected with approximately 0.2 µL bacterial suspension using a micromanipulator and microinjector (M-152 and IM-5B; Narishige) under a stereomicroscope (MZ75; Leica) inside a sterile bench (CleanAir). Images were taken using standard or confocal microscopy (Eclipse E600; Nikon, Chiyoda, Japan; or DMIL or SP5; Leica).

Statistical Analysis. For experiments stating p values, a paired Student's t-test was performed as described previously[5], assuming and stating significance at P 0.05 (*); P 0.01 (**) and P 0.005 (***).

SUPPLEMENTARY TABLES

Supplementary Table S1 | EDX analysis.

Samples	C	N	O	Na	Si	Ce
E. coli	64.11	12.06	21.73	1.01	0	0
***E. coli* + Si_{30}**	50.43	9.62	34.99	0.28	**3.98**	0

Supplementary Table S1. EDX analysis. Si was only detected in the sample treated with Si_{30}. Numbers are given in atomic percent.

Supplementary Table S2 | Gastric fluid (GF) simulation media

Components	GF
**Pepsin**	0.1 mg/mL
**Sodium taurocholate**	80 µM
**Lecithin**	20 µM
**Sodium chloride**	34.2 mM
**Orthophosphoric acid**	-
**Sodium dihydrogenphosphate**	-
**Milk/buffer**	1:1
**Hydrochloric acid**	for GF pH 3

Supplementary Table S2. Gastric fluid (GF) simulation media. Gastric fluid was used to simulate gastric conditions.

SUPPLEMENTARY FIGURES

Supplementary Fig. S1

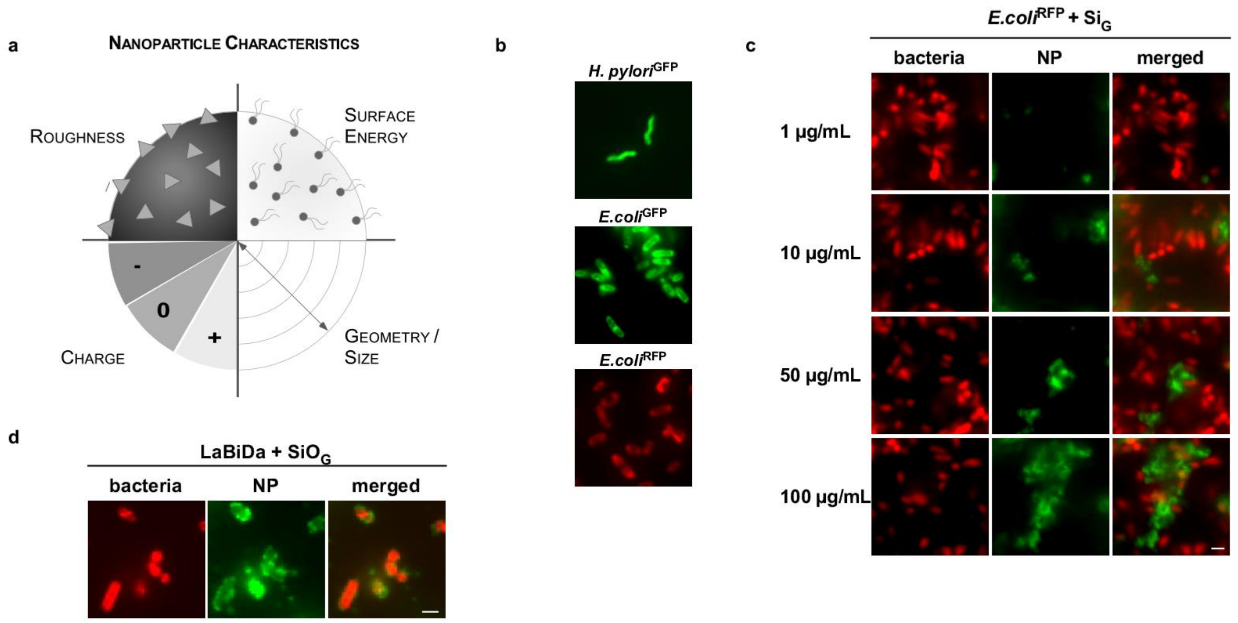

Supplementary Figure S1| Investigation of NP-bacteria complex formation. a, Physico-chemical characteristics of NMs' affecting their behavior and activity in physiological systems. **b**, Transgenic *E. coli* or *H. pylori* strains expressing red fluorescent protein tdTomato (here E. coli^{RFP}) or green fluorescent protein GFP (here: E. coli^{GFP}, *H. pylori*^{GFP}) were used to generate autofluorescent bacteria. Living bacteria can be visualized by fluorescence microscopy without additional staining. Scale bar 2 µm. **c**, Increasing amount of NPs results in higher numbers of NP-bacteria complexes *in situ*. Representative images of NP-bacteria complexes visualized by fluorescence microscopy. Scale bars, 2 µm. **d**, NP-binding was also found for so called 'probiotic' bacteria, e.g., *Lactobacillus acidophilus*, *Bifidobakterium lactis* and *Streptococcus thermophiles* (LaBiDa).

Supplementary Fig. S2

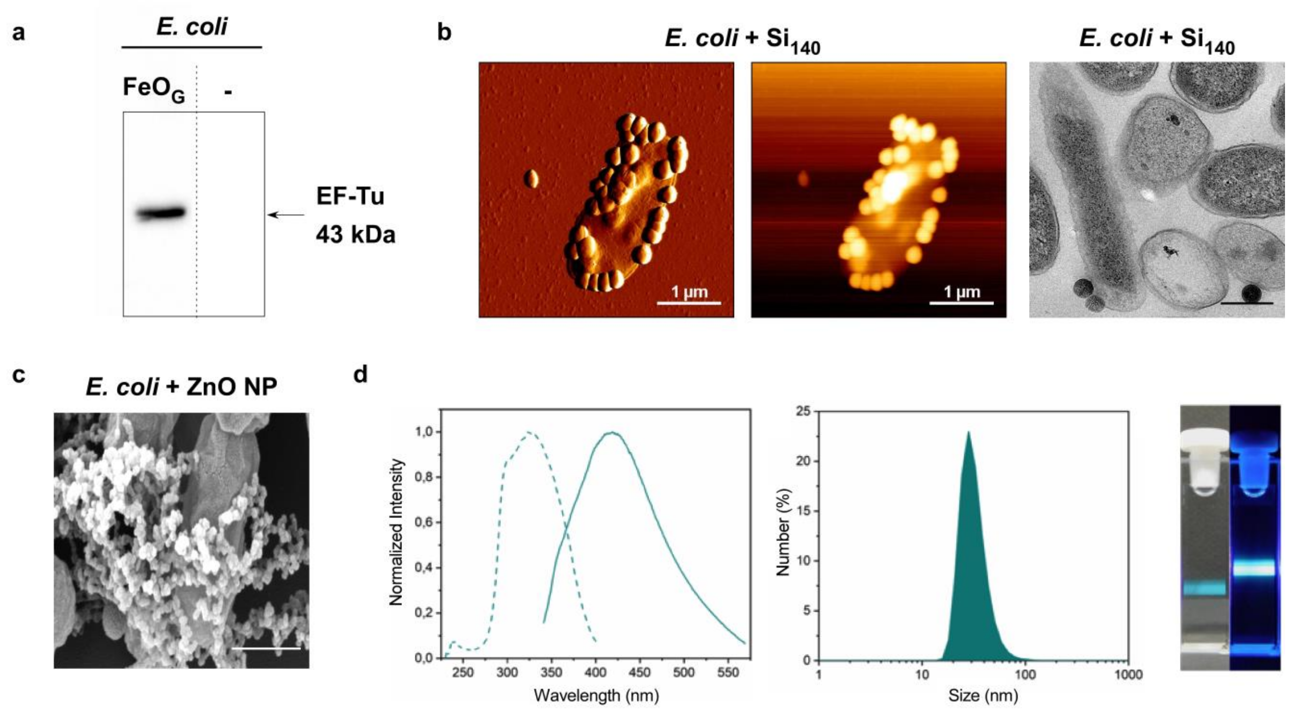

Supplementary Figure S2| NPs rapidly and stably adsorb to various pathogens under physiologically relevant conditions. a, Immunoblot analysis was performed after incubation of FeO_G-NPs and bacteria for 10 min in PBS and subsequent magnetic separation of FeO_G from the samples. The antibody used detected EF-Tu (elongation factor thermo unstable) of bacterial cells, thus confirming NP-bacteria complex formation. In samples without NP incubation, EF-Tu was not detectable. **b-c**, AFM, TEM, and SEM images of NP-bacteria complexes. Bacteria were incubated with indicated materials in PBS for 10 min. Scale bars, 500 nm. **d**, Excitation and emission spectra of isolated BNP (left). DLS measurement of the BNP (middle). Solution of BNP excited with a 405 nm laserpointer under daylight and in the dark to demonstrate blue autofluorescence (right).

Supplementary Fig. S3

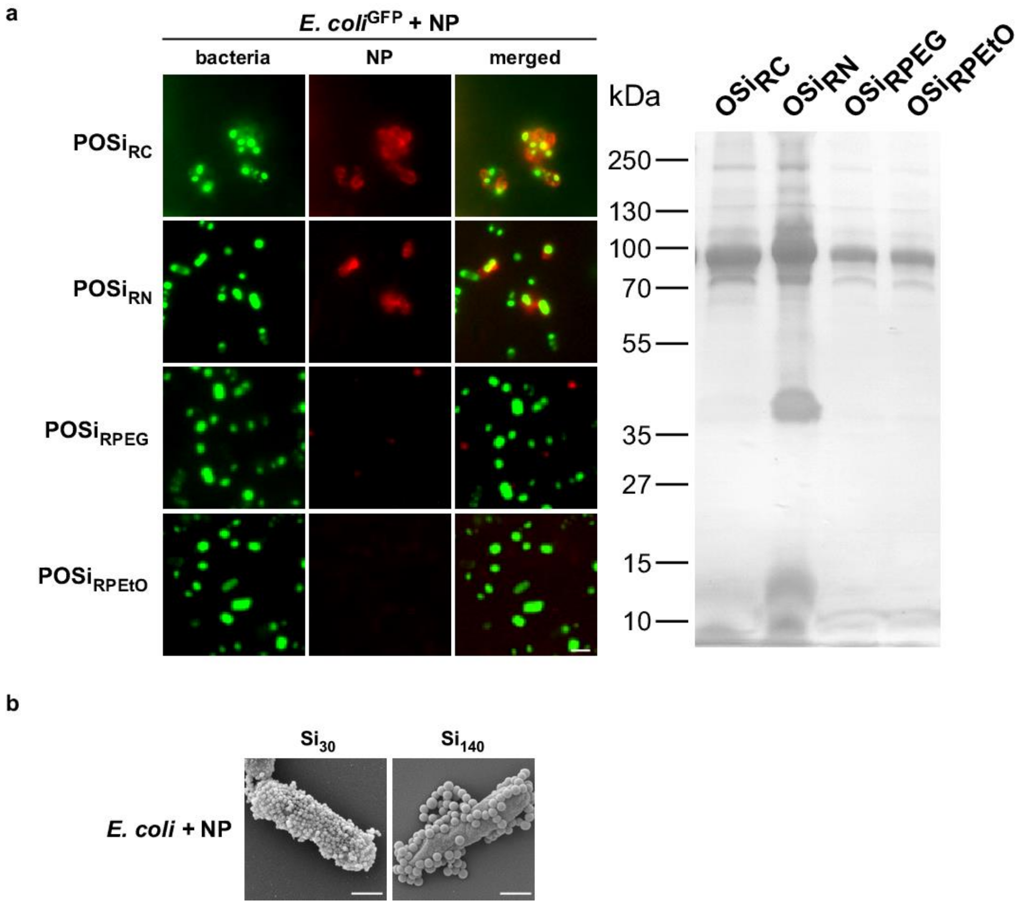

Supplementary Figure S3| NPs' physico-chemical characteristics determine binding to bacterial surface. a, Positively- (POSi$_{RN}$) and negatively- (POSi$_{RC}$) charged poly(organosiloxane) NPs bound to bacteria. Less binding was observed for the negatively charged NPs and no binding occurred for 'stealth'-modified NPs. All images are representatives of three independent experiments (left panel). SDS-PAGE demonstrates reduced overall plasma protein binding for 'stealth'-modified NPs by the addition of poly(ethylene glycol) or poly(2-ethyl-2-oxazoline). NPs were incubated in undiluted human plasma for 30 min and washed (right panel). **b**, SEM images of NP-bacteria complexes. Bacteria were incubated with indicated materials in PBS for 10 min. Scale bars, 500 nm.

Supplementary Figure S4

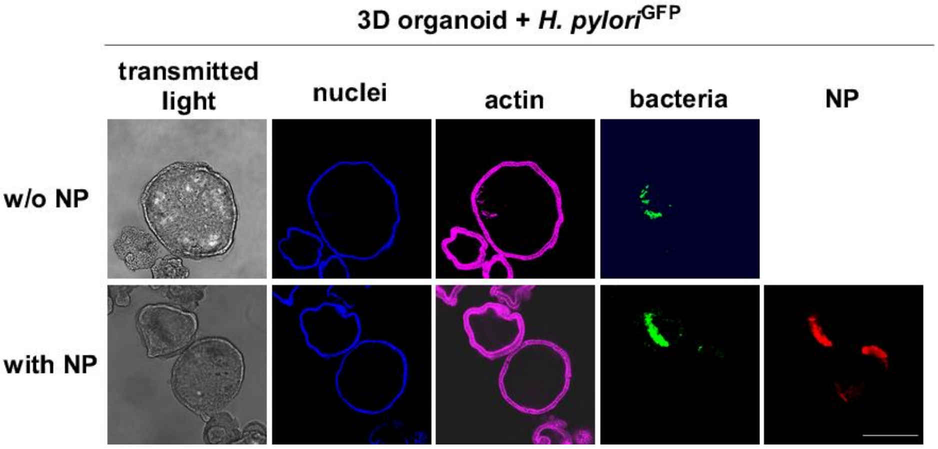

Supplementary Figure S4| NP-bacteria complexes attached to 3D organoid structures. 3D gastric organoids from healthy human corpus mucosa were infected with pristine bacteria or Si_R NP-*H. pylori*^{GFP} complexes and analyzed by confocal fluorescence microscopy 8 h later. Pristine as well as Si_R-*H. pylori* complexes attached to 3D organoid structures equally well. To prepare complexes $1x10^8$ *H. pylori* cells and 600 µg/mL Si_{30}-NP were incubated in PBS for 10 min, theoretically resulting in a 25 % coverage of the cell surface.

Supplementary Fig. S5

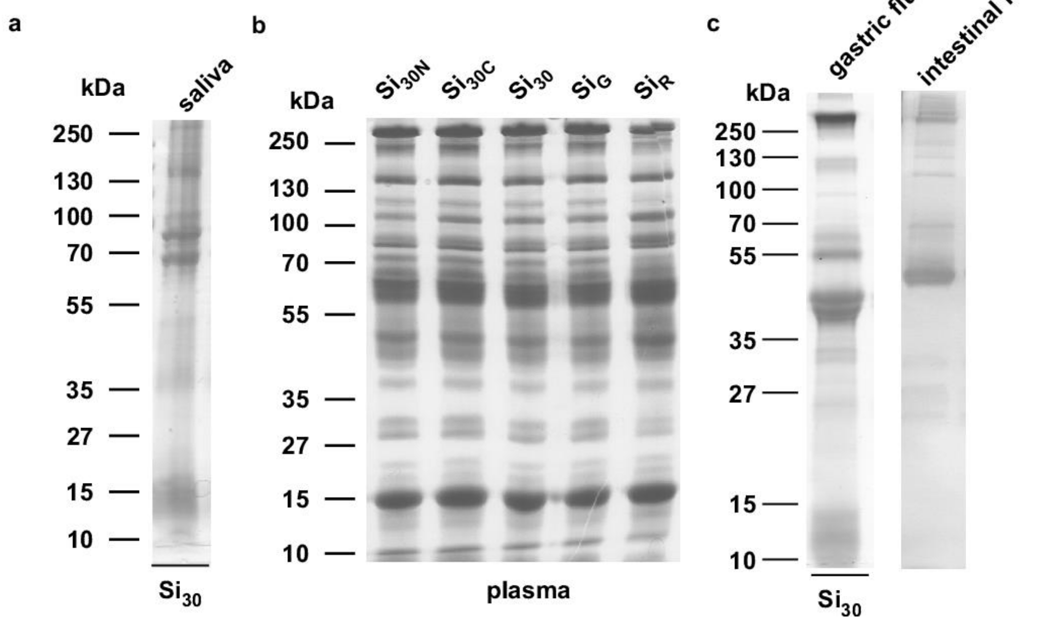

Supplementary Figure S5| SDS-PAGE demonstrates that all tested NPs acquire a protein corona. a-c Indicated NPs were incubated in the respective physiological fluid for 30 min, washed twice with PBS and bound corona proteins detached via heating in SDS sample buffer. Protein was visualized after separation via SDS gel electrophoresis by Coomassie staining.

Supplementary References

1. Docter, D.; Distler, U.; Storck, W.; Kuharev, J.; Wunsch, D.; Hahlbrock, A.; Knauer, S. K.; Tenzer, S.; Stauber, R. H., Quantitative profiling of the protein coronas that form around nanoparticles. *Nature protocols* **2014,** *9* (9), 2030-44.

2. Tenzer, S.; Docter, D.; Kuharev, J.; Musyanovych, A.; Fetz, V.; Hecht, R.; Schlenk, F.; Fischer, D.; Kiouptsi, K.; Reinhardt, C.; Landfester, K.; Schild, H.; Maskos, M.; Knauer, S. K.; Stauber, R. H., Rapid formation of plasma protein corona critically affects nanoparticle pathophysiology. *Nature nanotechnology* **2013,** *8* (10), 772-81.

3. Xiao, J.; Kuc, A.; Pokhrel, S.; Madler, L.; Pottgen, R.; Winter, F.; Frauenheim, T.; Heine, T., Fe-doped ZnO nanoparticles: the oxidation number and local charge on iron, studied by 57Fe Mossbauer spectroscopy and DFT calculations. *Chemistry* **2013,** *19* (10), 3287-91.

4. Koshkina, O.; Westmeier, D.; Lang, T.; Bantz, C.; Hahlbrock, A.; Wurth, C.; Resch-Genger, U.; Braun, U.; Thiermann, R.; Weise, C.; Eravci, M.; Mohr, B.; Schlaad, H.; Stauber, R. H.; Docter, D.; Bertin, A.; Maskos, M., Tuning the Surface of Nanoparticles: Impact of Poly(2-ethyl-2-oxazoline) on Protein Adsorption in Serum and Cellular Uptake. *Macromol Biosci* **2016**.

5. Tenzer, S.; Docter, D.; Rosfa, S.; Wlodarski, A.; Kuharev, J.; Rekik, A.; Knauer, S. K.; Bantz, C.; Nawroth, T.; Bier, C.; Sirirattanapan, J.; Mann, W.; Treuel, L.; Zellner, R.; Maskos, M.; Schild, H.; Stauber, R. H., Nanoparticle Size Is a Critical Physicochemical Determinant of the Human Blood Plasma Corona: A Comprehensive Quantitative Proteomic Analysis. *ACS nano* **2011,** *5* (9), 7155-7167.

6. Schmitt, W.; Haas, R., Genetic analysis of the Helicobacter pylori vacuolating cytotoxin: structural similarities with the IgA protease type of exported protein. *Mol Microbiol* **1994,** *12* (2), 307-19.

7. Sakulkhu, U.; Mahmoudi, M.; Maurizi, L.; Salaklang, J.; Hofmann, H., Protein corona composition of superparamagnetic iron oxide nanoparticles with various physico-chemical properties and coatings. *Scientific reports* **2014,** *4*, 5020.

8. Krisch, L. M.; Posselt, G.; Hammerl, P.; Wessler, S., CagA Phosphorylation in Helicobacter pylori-Infected B Cells Is Mediated by the Nonreceptor Tyrosine Kinases of the Src and Abl Families. *Infect Immun* **2016,** *84* (9), 2671-80.

9. Ritz, U.; Gotz, H.; Baranowski, A.; Heid, F.; Rommens, P. M.; Hofmann, A., Influence of different calcium phosphate ceramics on growth and differentiation of cells in osteoblast-endothelial co-cultures. *J Biomed Mater Res B Appl Biomater* **2016**.

10. Bartfeld, S.; Bayram, T.; van de Wetering, M.; Huch, M.; Begthel, H.; Kujala, P.; Vries, R.; Peters, P. J.; Clevers, H., In vitro expansion of human gastric epithelial stem cells and their responses to bacterial infection. *Gastroenterology* **2015,** *148* (1), 126-136 e6.

11. Sato, T.; Stange, D. E.; Ferrante, M.; Vries, R. G.; Van Es, J. H.; Van den Brink, S.; Van Houdt, W. J.; Pronk, A.; Van Gorp, J.; Siersema, P. D.; Clevers, H., Long-term expansion of epithelial organoids from human colon, adenoma, adenocarcinoma, and Barrett's epithelium. *Gastroenterology* **2011,** *141* (5), 1762-72.

12. Mangold, S.; Harneit, K.; Rohwerder, T.; Claus, G.; Sand, W., Novel combination of atomic force microscopy and epifluorescence microscopy for visualization of leaching bacteria on pyrite. *Appl Environ Microbiol* **2008,** 74 (2), 410-5.
